# Medical negligence compensation claims in knee meniscal surgery in Norway: a cross-sectional study

**DOI:** 10.1186/s13037-025-00427-1

**Published:** 2025-01-16

**Authors:** Frank-David Øhrn, Asbjørn Årøen, Tommy Frøseth Aae

**Affiliations:** 1 Møre and Romsdal Hospital Trust, Kristiansund Hospital, Kristiansund, Norway; 2 NTNU - Norwegian University of Science and Technology, Trondheim, Norway; 3 Institute of Clinical Medicine, University of Oslo, Oslo, Norway; 4 Akershus University Hospital, Lorenskog, Norway; 5 Oslo Sports Trauma Research Center, Oslo, Norway; 6 Department of Research and Innovation, Møre and Romsdal Hospital Trust, Kristiansund, Norway

**Keywords:** Compensation claims, Arthroscopic surgery, Meniscal surgery, Knee surgery, Gender differences

## Abstract

**Background:**

Meniscal surgery is one of the most frequent orthopaedic procedures performed worldwide. There is a wide range of possible treatment errors that can occur following meniscal surgery. In Norway, patients subject to treatment errors by hospitals and private institutions can file a compensation claim free of charge to the Norwegian System of Patient Injury Compensation (NPE). The purpose of this study was to systematically analyse compensation claims filed to the NPE following meniscal surgery and evaluate gender effects on accepted claims. Our hypothesis was that there was no gender difference in accepted claims.

**Methods:**

We performed a cross-sectional study assessing all registered claims at the NPE after meniscal surgery from 2010 to 2020. The surgical procedures were stratified into subgroups following data collection. Data from the Norwegian Patient Registry were collected to obtain information on the numbers of the different procedures performed in hospitals and private institutions. We calculated frequencies and relative frequencies of categorical data. Differences in categorical data were calculated using the Pearson Chi-square test.

**Results:**

The total number of meniscal resections and sutures in the study period was 119,528. A total of 372 compensation claims were filed, 241 male and 130 female. Of these, 152 (40.9%) claims were accepted, while 220 (59.1%) were rejected. The most frequent reasons for filing a compensation claim were pain (114), followed by infection (98), wrong technique (38) and impaired function/instability (25).There was a significant gender difference in the acceptance of claims in favour of men (121 vs. 31, *p* < 0.001). A sensitivity analysis excluding infection as reason for compensation claim found no gender difference (*p* = 0.16) in acceptance of claims.

**Conclusion:**

Compensation claims after meniscal surgery are rare, with only 0.3% of patients filing a compensation claim. There was a marked preponderance of men with accepted claims due to a higher frequency of postoperative infections. Surgeons should be aware of this and take this into account in the decision-making before surgery.

## Background

Meniscal surgery is one of the most common orthopaedic surgical procedures performed worldwide [1]. The most frequent meniscal surgeries are either arthroscopic sutures or (partial) resection of the meniscus. Open surgery is rare, as is transplantation of the meniscus.

In Norway, although most meniscal surgery is performed in public hospitals, there is also a significant number of surgeries in private healthcare facilities.

Treatment errors from meniscal resection and meniscal repair have been reported in between 2.8 and 11% of the cases [2, 3]. Treatment errors include but are not restricted to, chondral damage, pain, failure of suture device, infection, venous thromboembolism (VTE) and pulmonary embolism, nerve injuries (saphenous and peroneal) and popliteal artery lesions [4].

Patients in Norway experiencing treatment errors from private or public medical treatment can file a compensation claim free of charge to the Norwegian System of Patient Injury Compensation (NPE) [5]. Certain vital criteria must be present in order to receive compensation from the NPE: (1) The treatment received has to be incorrect according to NPE regulations, (2) the treatment error has to have financial consequences for the patient and (3) the claim has to be filed within 3 years of the treatment. However, if the error led to a level of impairment of more than 15%, compensation will in some cases be accepted even though the 3 criteria above are not fulfilled. This applies especially to postoperative infections. In such cases, the level of medical impairment is set by specialists pointed out by the NPE [6].

Compensation claims following medical treatment are common in orthopaedic surgery [7].There are no known nationwide registries for this specific procedure, like the ones we have in arthroplasties [8]. Using the registries of NPE and NPR therefore gives us access to a vast number of procedures that otherwise would be impossible to retrieve. Moreover, the compensation claims are subjective data, i.e. it is not the surgeon reporting these complaints or complications. Thus, in addition to the vast number of surgeries assessed, this gives us another perspective than experimental studies. Complications in orthopaedic surgery costs a lot for the society, in addition to the potential devastating results for the patients. However, a study on patient compensation claim is not evaluating complications after surgery, but identifying areas of improvement in health care. Studies on compensation claims therefore should be of global interest. Previous studies have assessed compensation claims following arthroplasty procedures, spine interventions, arthroscopic procedures in general, knee cartilage surgery and anterior cruciate ligament reconstruction [9–14]. To date, however, no study has examined compensation claims following the most frequently performed orthopaedic procedures, namely meniscal resection and meniscal suture. The aim of this study was to assess the frequency and distribution of compensation claims in Norway following knee meniscus surgical procedures and evaluate gender effects on accepted claims. The objective to retrieve and couple data of such surgeries from the NPE and NPR between 2010 and 2020. Our hypothesis was that there was no gender difference in accepted claims.

## Methods

This study has a cross-sectional design, and we assessed and evaluated *all* compensation claims for meniscal surgery in public and private healthcare facilities from 1 January 2010 to 31 December 2020. All patients who submitted a compensation claim following knee meniscal surgery to NPE in Norway during the study period were included.

The Norwegian Patient Registry (NPR) records *all* treatment in public and private Norwegian hospitals, and its main function is to collect and report data on patients in specialist care in Norway.

Data were collected from the NPE as the database was searched to identify patients by using predefined procedure codes based on the Nordic Medico-Statistical Committee (NOMESCO) Classification of Surgical Procedures (NCSP) codes (Table 1) [15]. No patients were excluded from the NPE database, which means that *all* of the patients that filed a complaint after surgery as depicted in Table 1, were included in the study data set. Data from the NPE included demographics, treatment facilities, procedure performed, the reason for the claim and the outcome (accepted/rejected). The surgical procedures were stratified into subgroups following data collection. The corresponding data from the NPR were collected and included information on the numbers of different procedures performed in public and private hospitals. All data were retrieved in late 2023.

**Table 1:** Nordic medico-statistical committee (NOMESCO) classification of surgical procedures (NCSP) [15]

Surgical procedure	NCSP code
NGD 01	Arthroscopic total excision of the meniscus
NGD 02	Open total excision of the meniscus
NGD 11	Arthroscopic partial excision of meniscus
NGD 12	Open partial excision of meniscus
NGD 21	Arthroscopic reinsertion of meniscus
NGD 22	Open reinsertion of meniscus
NGD 91	Other arthroscopic operation on meniscus
NGD 92	Other open operation on meniscus

The Regional Ethics Committee of Norway (REK) approval and consent from the patients were deemed unnecessary because the data are based on anonymized records (REK 15.10.10). In addition, the Data Access Committee and the data protection officer of Møre and Romsdal Hospital Trust approved the study on the 6th of February 2024 (approval number 2024/1390).

### Statistics

We calculated frequencies and relative frequencies of categorical data. Differences in categorical data were calculated using the Pearson Chi-square test. A p value of < 0.05 was regarded as statistically significant. All statistics were calculated using IBM SPSS version 29 (IBM Corp., Armonk, NY, USA).

## Results

**Fig. 1:**
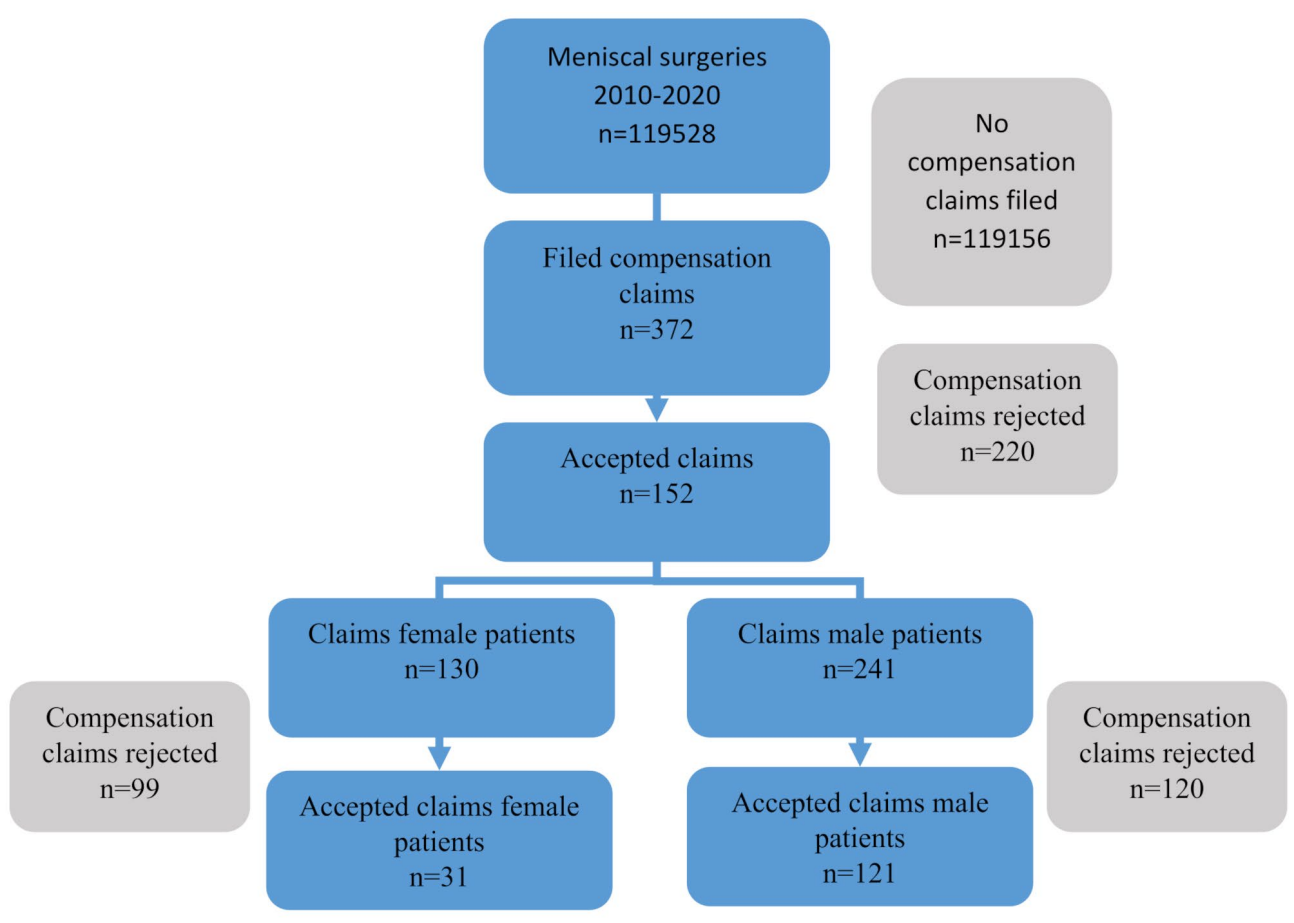
Flow chart of the compensation claims filed

The total number of meniscal resections and sutures in the study period was 119,528, of which almost 90% were meniscal resections (Fig. 1). A total of 372 (0.3%) compensation claims were filed during the study period (Tables 2 and 3). Of these, 152 (40.9%) claims were accepted, while 220 (59.1%) were rejected.

**Table 2:** Reasons for claims in declining order of frequency, stratified by gender.*In one of the cases, the gender of the patient was not reported

Compensation claim reason	Total		Accepted		Rejected		Percentage approved	
			Male	Female	Male	Female	Male	Female
Pain	114		1	1	56	56	2	2
Infection	98		82	9	6	1	93	90
Wrong technique	38		11	6	12	9	48	40
Impaired function/instability	25		2	0	16	7	11	0
Nerve lesion	22		2	1	12	7	14	13
Delayed treatment/surgery	21		5	2	4	10	56	17
Wrong or no indication	16		10	5	1	0	8	16
DVT or LE	13		0	2	8	3	0	40
Inadequate preop investigation	10		3	3	3	1	50	75
Iatrogenic damage	3		1	2	n/a	n/a	100	100
Development of osteoarthritis	2		n/a	n/a	1	1	0	0
Stiffness*	2		1	0	n/a	n/a	100	n/a
Operated wrong knee	2		1	0	0	1	50	0
Compartment syndrome	2		1	0	0	1	50	0
CRPS	2		n/a	n/a	n/a	2	n/a	0
Operation initiated rheumatoid arthritis	1		n/a	n/a	1	n/a	0	n/a
Death	1		1	n/a	n/a	n/a	100	n/a
Total	372		121	31	120	99	50	24

**Table 3:** Distribution of claims stratified by age, gender and accepted denied

Age	Male	Female	Unclassified	Accepted	Denied	Total
0–19	13	27	0	12	28	40
20–29	44	18	0	26	37	63
30–30	53	13	1	30	36	66
40–49	67	38	0	36	69	105
50–59	38	27	0	32	33	65
60–69	22	6	0	14	14	28
70–79	2	0	0	2	0	2
80+	0	1	0	0	1	1
Total	241	130	1	152	220	372

### Frequency of claims

There were 17 different reasons for filing compensation claims (Table 2). The most frequent reasons were pain (114) followed by infection (98), wrong technique (38) and impaired function/instability (25).

### Gender distribution of claims

Men filed 241 of the claims and women 130 (Table 3). 1 claim lacked information on gender. There was a significant gender difference in the acceptance of claims (*p* < 0.001) (Table 4). The numbers accepted were 121 (50%) for males and 31 (24%) for females. We therefore performed a sensitivity analysis (Table 4) where we removed infection as reason for compensation claim and then found no gender difference (*p* = 0.16).

**Table 4:** Sensitivity analysis of infection included and excluded stratified by gender

	Denied	Accepted	*P* value
Infection included			
Male	120	121	< 0.001
Female	99	31	
Infection excluded			
Male	114	39	0.16
Female	98	22	

### Age distribution of claims

**Fig. 2:**
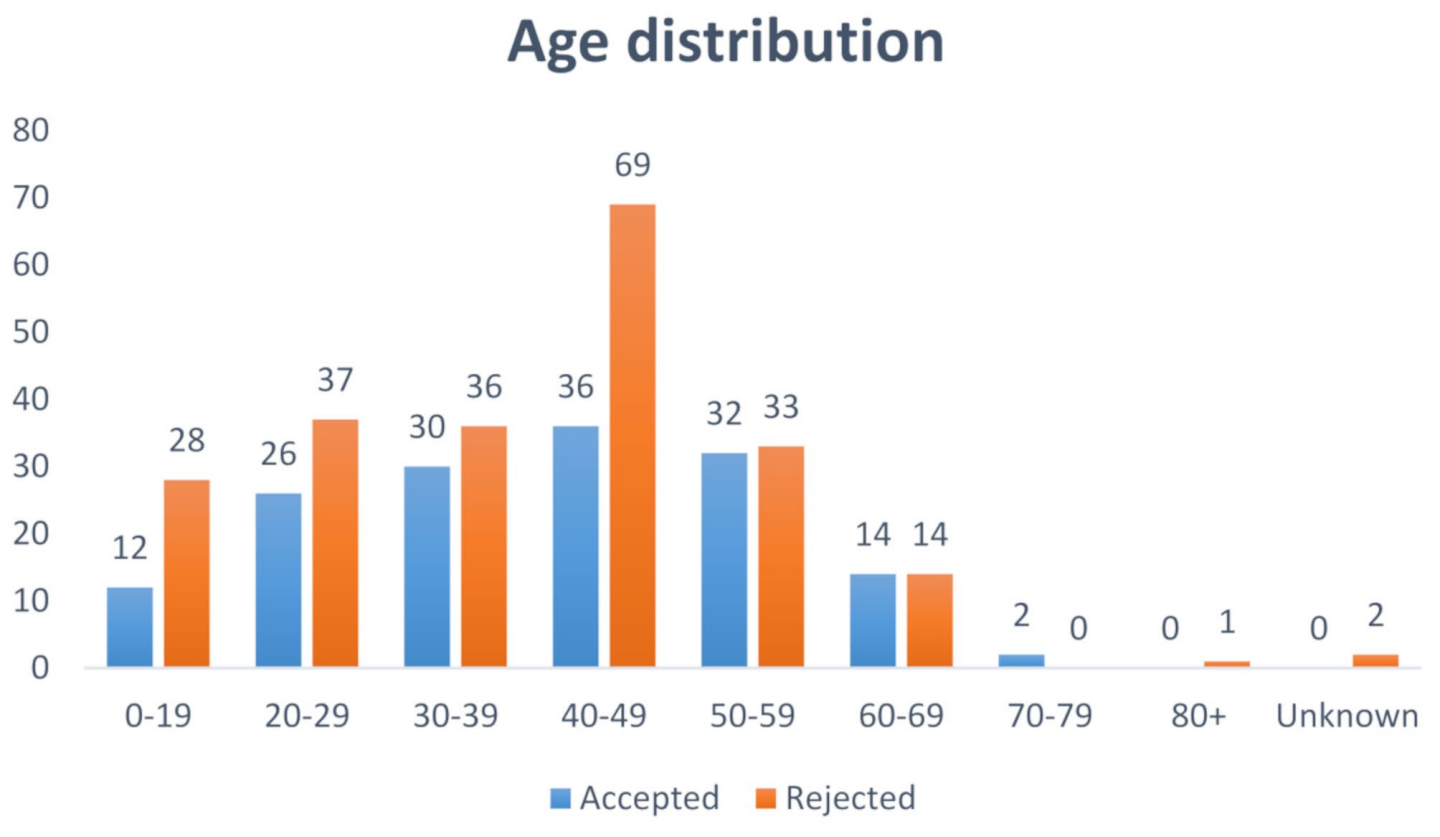
Age distribution of compensation claims

Most claims were filed by patients aged 20–60 years, with the highest frequency being in the age group 40–49 years. This age group filed 95 claims, 69 of which were rejected (Fig. 2). Stratifying into different age intervals (Table 3), there was no difference in accepted vs. rejected (*p* = 0.14) compensation claims.

### Relative frequency of acceptance

Although death (1) and iatrogenic damage (3) were very rare treatment errors among all the 119,528 surgeries, the filed claims were accepted in 100% of those cases. Wrong indication (16) and the more frequent infection (98) both resulted in compensation in 93% of the cases (Table 2).

### Meniscal resection versus meniscal repair

Most of the compensation claims were due to arthroscopic resections (284) and repair (80). These were accepted in 113 (40%) and 36 (45%) of the cases (*p* = 0.42).

## Discussion

The main finding of the present study was that the relative frequency of accepted claims for men was twice the figure for women. The number of compensation claims by males was almost 2/3 of the total number of claims. This can be explained by the fact that the frequency of compensation claims by men where the cause was infection was much higher than the corresponding frequency for women. One study has reported that men are more involved in shared decision-making processes than women. Despite this, our study reveals that men have more compensation claims accepted. Our sensitivity analysis, where we removed infection as a reason for compensation claims, showed that there was no gender difference in acceptance without infection as the cause (*p* = 0.16). The literature demonstrates that men have a higher risk of infection following meniscal surgery [16, 17]. It has been stated that this might be due to different baseline bacterial colonization of the skin by the two genders, and also that it might be due to more hair growth and thus need for shaving in men [18]. In the NPE system, compensation claims because of infection tend to be accepted, amounting to almost 93% in our database. This is consistent with other papers on the topic [9, 10, 13, 14].

It is important to assess and reinforce changes due to treatment failures. Incident reports and complaint management are important factors of the “continuous improvement cycle” [19]. We believe this applies to both hospital level and national levels. On a hospital level this can be taken care of with proper quality systems, and a culture for constructive managing of the complaints and treatment errors [19]. On a larger scale, registry based studies like ours can be important factors for reinforcing changes in treatment and decision-making.

In the study period, 0.3% of patients filed a compensation claim and 0.13% were accepted. Pain was the single most frequent reason for filing a compensation claim, with 114 claims among the more than 119,500 surgeries (0.1%). Pain is the cardinal symptom of early osteoarthritis (OA), and some of the surgeries might have been performed on early OA where a meniscus injury was associated with, or part of the process in, the aging knee [20, 21]. As outlined in the results of the present study, compensation claims were most frequent in the higher age groups, which supports this assumption. These patients received compensation in fewer than 2% of the cases. Pain is a rather subjective complication, and no tests can be performed to confirm this treatment error. It can be due to intra-articular damage, such as cartilage lesions, progression of osteoarthritis, complex regional pain syndrome, arthrofibrosis, etc. It can also be due to the original condition, i.e. the problem was not solved by the arthroscopic procedure. Recent literature recommends alternative approaches to surgery for a degenerative meniscus injury [22, 23]. Our data do not discriminate between the different causes of pain. However, iatrogenic damage and complex regional pain syndrome are separate reasons for compensation claims. The former claims were filed in 3 (0.003%) cases, which were all accepted, while the latter were filed in 2 (0.002%) cases and neither of these was accepted. Many articles on complications of meniscal surgery do not report pain as a complication. Hagino et al. did not see any cases of complex regional pain syndrome in a study of more than 2.600 cases. 2 out of these cases did, however, develop postoperative infection, and 2 developed VTE [24]. Pajalic et al. published in 2018 a comprehensive article on 18,735 patients who underwent knee arthroscopy in Sweden [25]. They found an overall complication rate of 1.1%. The three most reported complications were complications not classified elsewhere (33%), thrombophlebitis (24%) and pulmonary embolism (14%) [25]. However, they only focused on complications in the 30 days following arthroscopic procedures in the knee [25]. This differs from our results, which show that 13 (0.01%) compensation claims were filed because of VTE or pulmonary embolism 2 of which (15%) were accepted.

Wrong technique was the third most frequent reason for compensation claims in our data. 38 (0.03%) compensation claims were filed for this reason, and they were accepted in 45% of the cases. Our data do not reveal whether the errors were caused by inexperienced surgeons, who either did not use the technique correctly or chose the incorrect technique at the outset. Examples could be that the surgeon tried to use cortical fixation for a meniscal root tear but placed the suture channels incorrectly, or that the surgeon did not realize that there was a root tear in the first place, and therefore did not address it. Nevertheless, little has been written about wrong techniques. Inexperience can also lead to higher surgical time, which increases the likelihood of complications. In 2006, Reigstad et al. found a complication rate of 5% in 876 cases, but only 0.7% had therapeutic consequences. In their data they found that the time spent on the procedure was the only factor that influenced the rate of complications, thus not the use of a tourniquet [26]. In a larger study by Gowd et al. of almost 79,000 arthroscopic knee surgeries, only small increases in surgical time increased the risk of complications such as postoperative infections, sepsis and readmissions [27].

Impaired function/instability and nerve lesion complaints were filed in 25 (0.02%) and 22 (0.02%) cases respectively. The patients received compensation in 8% and 14% of the cases.

### Delayed treatment/surgery

21 patients (0.02%) filed a claim for compensation for delayed treatment or surgery, and 7 (33%) of these were successful. This seems reasonable, as many meniscal tears are chronic. A patient with a long relevant medical history is not likely to benefit from faster treatment, and according to Pihl et al., there is no subgroup that benefits more from meniscal surgery than others [28]. In addition, shorter time to surgery was not a success factor [28]. It seems, however, reasonable to treat patients with acute trauma more promptly. When a repair of the meniscus is indicated, it is advised to do this as soon as possible [29]. This can explain the figure of 33% who received compensation in this group.

### Wrong or no indication

16 (0.01%) patients filed a claim for this reason, and almost all of them (93%) were granted compensation. The mean age and volume of meniscal resection continued to rise in this period [30, 31], despite the questionable results of arthroscopy for degenerative knees [32, 33]. The study of Kise et al. indicates that one can achieve good results with supervised exercise alone in this group of patients [34]. This may be the reason why such a high percentage received compensation in this group.

### Limitations of the study

In Norway, there is unfortunately no registry based on meniscal surgery. This means that we cannot determine whether our findings from the NPE are caused by different frequencies of filing complaints based on gender, or whether there actually is a difference in complications between the genders. However, other papers support the findings of gender differences [9, 10, 13, 14, 35], especially with regard to infection. Another limitation is the criteria for the NPE to accept compensation claims. Studies show that we can expect more complications than the 0.13% of accepted claims in the NPE [2, 36]. The vast difference in frequency between the types of claims accepted may also in turn affect whether people bother to file a claim or not, i.e. because compensation for infections is more often granted than for pain, people experiencing pain as a treatment error after meniscal surgery might be less likely to file a compensation claim in the first place. Further, the NCSP classification does not discriminate between the different types of meniscal sutures. We therefore have no data on possible differences between all-inside, inside-out and outside-in techniques in cortical sutures for meniscal root tears. However, the literature suggests that there is no difference [3]. Also, our study only included patients going through surgery, and not other treatment options such as physiotherapy or knee joint injections. All the patients in the study were all from one single country, and this may of course affect the generalizability of the study. Finally, being a cross-sectional study, we cannot perform causal inference. Such studies may also be susceptible to bias [37].

### Strengths of the study

The main strength of this study is that we examined more than 119,000 procedures over a period of 10 years. To our knowledge, there are no studies outnumbering the present study with respect to included subjects on compensation claims on meniscal knee surgery. In addition, the NPR and NPE registries have high consistency and completeness, and patients are unable to opt out. Hence, the figures are trustworthy [38] and reflect current practice and risk with this common knee surgery procedure. Collection of data on inferior outcomes is a powerful tool to study weak aspects of our current care in all fields of medicine and in this particular study meniscal surgery is in focus. This is valuable information for both knee surgeons and caregivers, and can be used to prevent treatment errors in the future. Our data suggest that additional precautionary measures should be taken when operating on men. Although the relative frequency of infection is low in both men and women, our data indicate a much higher relative frequency of infection following knee meniscal surgery in male patients. This study demonstrates the importance of reducing treatment errors when providing health care and to prevent to such errors to occur.

## Conclusion

Compensation claims after meniscal surgery are rare, with only 0.3% of patients filing a compensation claim. There was a marked preponderance of men with accepted claims due to a higher frequency of postoperative infections. Surgeons should be aware of this and take this into account in the decision-making before surgery.

## Data Availability

No datasets were generated or analysed during the current study.

## Abbreviations

DAC: Data access committee of Møre and Romsdal Hospital Trust
NCSP: Nordic Medico-Statistical Committee (NOMESCO) Classification of Surgical Procedures
NOMESCO: Nordic Medico-Statistical Committee
NPE: Norwegian System of Patient Injury Compensation
NPR: The Norwegian Patient Registry
OA: Osteoarthritis
REK: The Regional Ethics Committee of Norway
VTE: Venous thromboembolism

## References

[1] Vasta S, Papalia R, Albo E, Maffulli N, Denaro V. Top orthopedic sports medicine procedures. J Orthop Surg Res. 2018;13(1):190. 30064451 10.1186/s13018-018-0889-8 PMC6069744

[2] Salzler MJ, Lin A, Miller CD, Herold S, Irrgang JJ, Harner CD. Complications after arthroscopic knee surgery. Am J Sports Med. 2014;42(2):292–6. 24284049 10.1177/0363546513510677

[3] Fillingham YA, Riboh JC, Erickson BJ, Bach BR Jr., Yanke AB. Inside-out Versus All-Inside repair of isolated meniscal tears: an updated systematic review. Am J Sports Med. 2017;45(1):234–42. 26989072 10.1177/0363546516632504

[4] Taylor MZ, Caldwell PE 3rd, Pearson SE. Failure and complication rates in Common sports and arthroscopic procedures: reality check. Sports Med Arthrosc Rev. 2022;30(1):10–6. 35113837 10.1097/JSA.0000000000000338

[5] No authors listed. Norsk Pasientskade Erstatning (NPE). Avaliable at https://www.npe.no/no/Erstatningssoker/Soke-erstatning/Hva-skal-til-for-aa-faa-erstatning-pasientskade/ (Date assessed June 7, 2024).

[6] Forskrift om menerstatning ved yrkesskade, Lovdata. Available at https://lovdata.no/dokument/SF/forskrift/1997-04-21-373/KAPITTEL_2#KAPITTEL_2 (Date assessed June 13, 2024).

[7] Jena AB, Seabury S, Lakdawalla D, Chandra A. Malpractice risk according to physician specialty. N Engl J Med. 2011;365(7):629–36. 21848463 10.1056/NEJMsa1012370 PMC3204310

[8] No authors listed. Australian Orthopaedic Association National Joint Replacement Registry (AOANJRR). Available at https://aoanjrr.sahmri.com/annual-reports-2023 (Date assessed June 7, 2024).

[9] Randsborg PH, Aae TF, Bukholm IRK, Fenstad AM, Furnes O, Jakobsen RB. Compensation claims after knee arthroplasty surgery in Norway 2008–2018. Acta Orthop. 2021;92(2):189–93. 33439091 10.1080/17453674.2020.1871187 PMC8158226

[10] Aae TF, Jakobsen RB, Bukholm IRK, Fenstad AM, Furnes O, Randsborg PH. Compensation claims after hip arthroplasty surgery in Norway 2008–2018. Acta Orthop. 2021;92(3):311–5. 33459568 10.1080/17453674.2021.1872901 PMC8231378

[11] Debono B, Gerson C, Le Moing V, Houselstein T, Bougeard R, Lonjon G, Lonjon N. Spine surgery infection, litigation, and Financial Compensation: analysis of 98 claims involving French spine surgeons between 2015 and 2019. World Neurosurg. 2022;159:e161–71. 34902601 10.1016/j.wneu.2021.12.022

[12] Randsborg PH, Bukholm IRK, Jakobsen RB. Compensation after treatment for anterior cruciate ligament injuries: a review of compensation claims in Norway from 2005 to 2015. Knee Surg Sports Traumatol Arthrosc. 2018;26(2):628–33. 29181559 10.1007/s00167-017-4809-y PMC5794839

[13] Aae TF, Lian ØB, Årøen A, Engebretsen L, Randsborg PH. Compensation claims after knee cartilage surgery is rare. A registry-based study from Scandinavia from 2010 to 2015. BMC Musculoskelet Disord. 2020;21(1):287. 32384890 10.1186/s12891-020-03311-4 PMC7206764

[14] Lian OM, Randsborg PH, Jakobsen RB, Khan Bukholm IR, Aae TF. Prevalence of malpractice claims after arthroscopic shoulder surgery: analysis of 69,097 procedures from a national registry in Norway. Patient Saf Surg. 2023;17(1):25. 37853493 10.1186/s13037-023-00378-5 PMC10585783

[15] No authors listed. NOMESCO Classification of Surgical Procedures. Avaliable at https://norden.diva-portal.org/smash/get/diva2:970547/FULLTEXT01.pdf(Date assessed June 7, 2024).

[16] Balato G, Di Donato SL, Ascione T, D’Addona A, Smeraglia F, Di Vico G, Rosa D. Knee septic arthritis after arthroscopy: incidence, risk factors, functional outcome, and infection eradication rate. Joints. 2017;5(2):107–13. 29114639 10.1055/s-0037-1603901 PMC5672874

[17] Yeranosian MG, Petrigliano FA, Terrell RD, Wang JC, McAllister DR. Incidence of postoperative infections requiring reoperation after arthroscopic knee surgery. Arthroscopy. 2013;29(8):1355–61. 23906274 10.1016/j.arthro.2013.05.007

[18] Cohen B, Choi YJ, Hyman S, Furuya EY, Neidell M, Larson E. Gender differences in risk of bloodstream and surgical site infections. J Gen Intern Med. 2013;28(10):1318–25. 23605308 10.1007/s11606-013-2421-5 PMC3785652

[19] Moldovan F, Moldovan L. Assessment of patient matters in Healthcare Facilities. Healthc (Basel). 2024;12(3). 10.3390/healthcare12030325 PMC10855928 38338210

[20] Madry H, Kon E, Condello V, Peretti GM, Steinwachs M, Seil R, et al. Early osteoarthritis of the knee. Knee Surg Sports Traumatol Arthrosc. 2016;24(6):1753–62. 27000393 10.1007/s00167-016-4068-3

[21] Thorstensson CA, Andersson ML, Jönsson H, Saxne T, Petersson IF. Natural course of knee osteoarthritis in middle-aged subjects with knee pain: 12-year follow-up using clinical and radiographic criteria. Ann Rheum Dis. 2009;68(12):1890–3. 19054828 10.1136/ard.2008.095158

[22] Siemieniuk RAC, Harris IA, Agoritsas T, Poolman RW, Brignardello-Petersen R, Van de Velde S, et al. Arthroscopic surgery for degenerative knee arthritis and meniscal tears: a clinical practice guideline. BMJ. 2017;357:j1982. 28490431 10.1136/bmj.j1982 PMC5426368

[23] Beaufils P, Becker R, Kopf S, Matthieu O, Pujol N. The knee meniscus: management of traumatic tears and degenerative lesions. EFORT Open Rev. 2017;2(5):195–203. 28698804 10.1302/2058-5241.2.160056 PMC5489759

[24] Hagino T, Ochiai S, Watanabe Y, Senga S, Wako M, Ando T, et al. Complications after arthroscopic knee surgery. Arch Orthop Trauma Surg. 2014;134(11):1561–4. 25047161 10.1007/s00402-014-2054-0

[25] Friberger Pajalic K, Turkiewicz A, Englund M. Update on the risks of complications after knee arthroscopy. BMC Musculoskelet Disord. 2018;19(1):179. 29859074 10.1186/s12891-018-2102-y PMC5984803

[26] Reigstad O, Grimsgaard C. Complications in knee arthroscopy. Knee Surg Sports Traumatol Arthrosc. 2006;14(5):473–7. 16208459 10.1007/s00167-005-0694-x

[27] Gowd AK, Liu JN, Bohl DD, Agarwalla A, Cabarcas BC, Manderle BJ, et al. Operative time as an independent and modifiable risk factor for short-term complications after knee arthroscopy. Arthroscopy. 2019;35(7):2089–98. 31227396 10.1016/j.arthro.2019.01.059

[28] Pihl K, Ensor J, Peat G, Englund M, Lohmander S, Jørgensen U, et al. Wild goose chase - no predictable patient subgroups benefit from meniscal surgery: patient-reported outcomes of 641 patients 1 year after surgery. Br J Sports Med. 2020;54(1):13–22. 31186258 10.1136/bjsports-2018-100321

[29] Kopf S, Beaufils P, Hirschmann MT, Rotigliano N, Ollivier M, Pereira H, et al. Management of traumatic meniscus tears: the 2019 ESSKA meniscus consensus. Knee Surg Sports Traumatol Arthrosc. 2020;28(4):1177–94. 32052121 10.1007/s00167-020-05847-3 PMC7148286

[30] Degen RM, Lebedeva Y, Birmingham TB, Marsh JD, Getgood AMJ, Giffin JR, et al. Trends in knee arthroscopy utilization: a gap in knowledge translation. Knee Surg Sports Traumatol Arthrosc. 2020;28(2):439–47. 31359100 10.1007/s00167-019-05638-5

[31] Thorlund JB, Hare KB, Lohmander LS. Large increase in arthroscopic meniscus surgery in the middle-aged and older population in Denmark from 2000 to 2011. Acta Orthop. 2014;85(3):287–92. 24800623 10.3109/17453674.2014.919558 PMC4062797

[32] Thorlund JB, Juhl CB, Roos EM, Lohmander LS. Arthroscopic surgery for degenerative knee: systematic review and meta-analysis of benefits and harms. BMJ. 2015;350:h2747. 26080045 10.1136/bmj.h2747 PMC4469973

[33] Järvinen TL, Sihvonen R, Englund M. Arthroscopy for degenerative knee–a difficult habit to break? Acta Orthop. 2014;85(3):215–7. 24847793 10.3109/17453674.2014.922736 PMC4062784

[34] Kise NJ, Risberg MA, Stensrud S, Ranstam J, Engebretsen L, Roos EM. Exercise therapy versus arthroscopic partial meniscectomy for degenerative meniscal tear in middle aged patients: randomised controlled trial with two year follow-up. BMJ. 2016;354:i3740. 27440192 10.1136/bmj.i3740 PMC4957588

[35] Hoseth JM, Aae TF, Jakobsen RB, Fenstad AM, Bukholm IRK, Gjertsen JE, Randsborg PH. Compensation claims after hip fracture surgery in Norway 2008–2018. Geriatr Orthop Surg Rehabil. 2023;14:21514593231188623. 37435443 10.1177/21514593231188623 PMC10331336

[36] Marmor S, Farman T, Lortat-Jacob A. Joint infection after knee arthroscopy: medicolegal aspects. Orthop Traumatol Surg Res. 2009;95(4):278–83. 19524495 10.1016/j.otsr.2009.04.009

[37] Wang X, Cheng Z, Cross-Sectional Studies. Strengths, weaknesses, and recommendations. Chest. 2020;158(1s):S65–71. 32658654 10.1016/j.chest.2020.03.012

[38] Bakken IJNK, Halsteinli V, Huse UH. and, FE S. Norsk pasientregister: Administrativ database med mange forskningsmuligheter. Norsk Epidemiologi 14(1). 2004.

